# Health targets in the Preventive Health Care Act and their importance for prevention reporting

**DOI:** 10.17886/RKI-GBE-2017-091

**Published:** 2017-08-30

**Authors:** Ulrike Maschewsky-Schneider

**Affiliations:** Chair evaluation committee of gesundheitsziele.de Gesellschaft für Versicherungswissenschaft und -gestaltung e. V. (GVG)

## Abstract

Experiences with the evaluation concept developed by the German forum *gesundheitsziele.de* (health targets) and the results obtained should be taken into consideration during the implementation of prevention reporting. Besides selecting and developing (sub-) targets, evaluating target achievement (impact evaluation) is an important aspect of the concept. This article discusses the methodological challenges for impact evaluation in addition to providing approaches to solve these issues.

## The German health targets forum gesundheitsziele.de

is a forum that consists of federal and federal state representatives, associations of the German health system and other non-governmental organisations. The collaboration is based on the principle of consensus. The members jointly develop national health targets, recommend measures to achieve the set goals and present recommendations for the practical implementation of measures. The forum ensures the results are made public and encourages the decentralised implementation of national health targets by stakeholders. The forum regularly evaluates and optimises target achievement rates and processes [1]. Germany’s Preventive Health Care Act underpins published national health targets as well as prevention reporting according to Book V of the Social Code SGB (§ 20d (4)(1-3)) and the scientific evaluation and measurement of target achievement according to Book V of the SGB (SGB V, § 20 (2)(1)) [2].

## The evaluation concept

Experiences with the evaluation concept, which was developed and implemented by academic experts, and the results it provides can be of use for prevention reporting. It is built around the following four areas:

I. Target selection: Members of the health target forum name relevant fields of interest that are compiled to be assessed on the basis of a scientific catalogue of criteria [3]. Based on this information, members of the forum agree on the selection and prioritisation of the targets they intend to develop in detail.

II. Workgroups are established to elaborate the targets and sub-targets, guided by the following key points:

► Targets are directed at the population and the health and social systems and aim to respect the principles of equal opportunity (gender, migration, socio-economic status) [4]
► They are based on the results of the scientific criteria analysis [3]
► Measures recommended to achieve targets should be evidence-based, their implementation feasible and impact measurable
► Criteria to quantify targets are also available

III. Monitoring (sub-) target achievement will be based on the following questions: Has the (sub-) target been achieved? Which measures for target achievement have been implemented and how have they contributed to the achievement of targets? What conclusions do the results allow regarding the need to update targets?

The complexity of measures provides methodological challenges at the different intervention levels which differ in extent and form. A broad range of stakeholders (such as service providers, stakeholders at the municipal level or private businesses) help achieve targets in diverse contexts (for example, settings and/or regions). Information is often scarce, in particular, concerning the effects of individual measures, the timespan during which such effects are visible and the mechanisms behind such effects, as well as the interplay between different measures in the achievement of targets.

Possible steps to limit these methodological challenges include:

► Selection of sub-targets and initial measures (priority measures) for evaluation
► Focus on the national level (structures and population) or on larger units (such as settings or particular health care sectors)
► Development of measurable indicators to monitor the implementation and effectiveness of initial measures at different levels
► Use of established sources of data
► Detection of gaps in the data (also over time)
► Identification of best practice models and relevant individual measures (particularly at the structural level)
► Theoretically grounded assumptions on the mechanisms that lead measures to be effective

IV. Overall evaluation: a survey of the participating stakeholders was undertaken in 2013 that enquired about their experiences with and expectations of the process we had installed to establish targets. The survey focused on the strategies and model projects that members had developed to initiate and implement the established health targets and on recommendations on how to enhance the additional value provided by the forum. 49 out of the 69 surveyed stakeholders had defined fields of action for their organisation that were related to national health targets. 54 stakeholders could provide at least one example of a measure to implement these health targets [5].

## Summary

The forum *gesundheitsziele.de* suggests that the experiences and results of the process to establish targets will be used by prevention reporting and that the evaluation committee and its expert academic members will thereby play an advisory role. Moreover, the results, methods and data that prevention reporting produces should be made available to the health target forum. We believe we should pursue the approach of impact evaluation and promote the methodological discourse on the evaluation of complex interventions as it is currently being discussed in public health research.
